# Domain-specific modulatory effects of phosphomimetic substitutions on liquid-liquid phase separation of tau protein

**DOI:** 10.1016/j.jbc.2023.104722

**Published:** 2023-04-17

**Authors:** Solomiia Boyko, Witold K. Surewicz

**Affiliations:** Department of Physiology and Biophysics, Case Western Reserve University, Cleveland, Ohio, USA

**Keywords:** amyloid, liquid-liquid phase separation, neurodegenerative diseases, protein aggregation, tau protein

## Abstract

Aggregation of tau is one of the major pathogenic events in Alzheimer’s disease and several other neurodegenerative disorders. Recent reports demonstrated that tau can condense into liquid droplets that undergo time-dependent transition to a solid-like state, suggesting that liquid condensates may be on the pathway to pathological aggregation of tau. While hyperphosphorylation is a key feature of tau isolated from brains of patients with Alzheimer’s disease and other tauopathies, the mechanistic role of phosphorylation in tau liquid-liquid phase separation (LLPS) remains largely unexplored. In an attempt to bridge this gap, here we performed systematic studies by introducing phosphomimetic substitutions of Ser/Thr residues with negatively charged Asp/Glu residues in different regions of the protein. Our data indicate that the phosphorylation patterns that increase the polarization of charge distribution in full-length tau (tau441) promote protein LLPS, whereas those that decrease charge polarization have an opposite effect. Overall, this study further supports the notion that tau LLPS is driven by attractive intermolecular electrostatic interactions between the oppositely charged domains. We also show that the phosphomimetic tau variants with low intrinsic propensity for LLPS can be efficiently recruited to droplets formed by the variants with high LLPS propensity. Furthermore, the present data demonstrate that phosphomimetic substitutions have a major effect on time-dependent material properties of tau droplets, generally slowing down their aging. The latter effect is most dramatic for the tau variant with substitutions within the repeat domain, which correlates with the decreased fibrillation rate of this variant.

The histopathological hallmark of a number of neurodegenerative disorders, such as Alzheimer’s disease (AD), Pick’s disease, frontotemporal dementia with parkinsonism-17, and progressive supranuclear palsy, is the presence of fibrillar aggregates of tau protein in brain (1, 2, 3, 4, 5, 6, 7, 8). These aggregates are toxic and have the ability to spread between cells by the prion-like mechanism (5, 6, 7, 8).

The structure of tau may be divided into several domains, including the N-terminal projection domain, the proline-rich domain (PRD), the pseudorepeat domain (RD), and the C-terminal domain (CTD) (5, 7). The protein is generally considered to be structurally disordered, even though elements of ordered secondary structure have been reported within the RD (7, 9). Due to an alternative splicing of the *MAPT* gene that encodes tau, the protein exists in at least six isoforms that contain different number of N-terminal inserts and/or different number of pseudorepeat sequences (5, 7).

Tau is known to undergo great many posttranslational modifications (5, 7, 10). An especially important of these is phosphorylation, as it not only regulates microtubule assembly but is also believed to modulate tau aggregation and contribute to disease pathology (11, 12). At least 85 potential phosphorylation sites have been identified in tau, most of which are readily accessible to kinases due to the tau’s largely unordered structure (5, 7, 10, 11, 12). Phosphorylation of approximately 45 of these sites has been observed in AD brain, most of which are serine and threonine residues in the PRD and CTD (13, 14, 15). Since AD tau contains on average ∼10 phosphates per molecule (with a distribution of phosphorylated sites between 4 and 19 per molecule) (14), there is a very large number of tau subpopulations with distinct phosphorylation patterns (14, 15). Furthermore, even though accumulation of hyperphosphorylated tau appears to be one of the main histopathological features in AD and other tauopathies, mechanistic aspects of the relationship between phosphorylation and protein aggregation in disease are not well understood.

It has been recently shown that tau readily undergoes liquid-liquid phase separation (LLPS) in the test tube (16, 17, 18, 19, 20, 21, 22) and in cells (18, 23, 24, 25, 26, 27, 28), and that condensation of tau into liquid droplets has a major regulatory effect on its aggregation (18, 22, 29, 30, 31). The propensity of full-length tau (tau441) for LLPS was found to diminish with increasing salt concentrations (20, 22), and recent studies indicate that intermolecular electrostatic interactions between the oppositely charged N-terminal and middle regions of the protein are the major driver of this reaction (20, 32).

It has been also reported that tau LLPS is affected by phosphorylation (18, 33, 34). However, the mechanism of this effect is poorly understood, and studies in this regard are complicated since tau isolated from brain or recombinantly expressed in mammalian of insect cells contains a mixture of many isoforms with distinct phosphorylation patterns. In an effort to overcome this difficulty, here we used a phosphomimetic approach which allowed us to introduce, in a systematic way, phosphomimetic amino acid substitutions in different parts of tau protein. Our data show that, as predicted by the electrostatic interactions model of tau LLPS, phosphomimetic substitution patterns that increase the polarization of charge distribution promote tau condensation, whereas those that decrease charge distribution polarization have the opposite effect. Furthermore, we show that the phosphomimetic variants with low intrinsic propensity for LLPS can be efficiently recruited to droplets formed by the variants with high LLPS propensity, and that phosphomimetic substitutions have a major effect on time-dependent material properties of tau droplets.

## Results

### Design of the phosphomimetic tau441 variants

**Figure 1:**
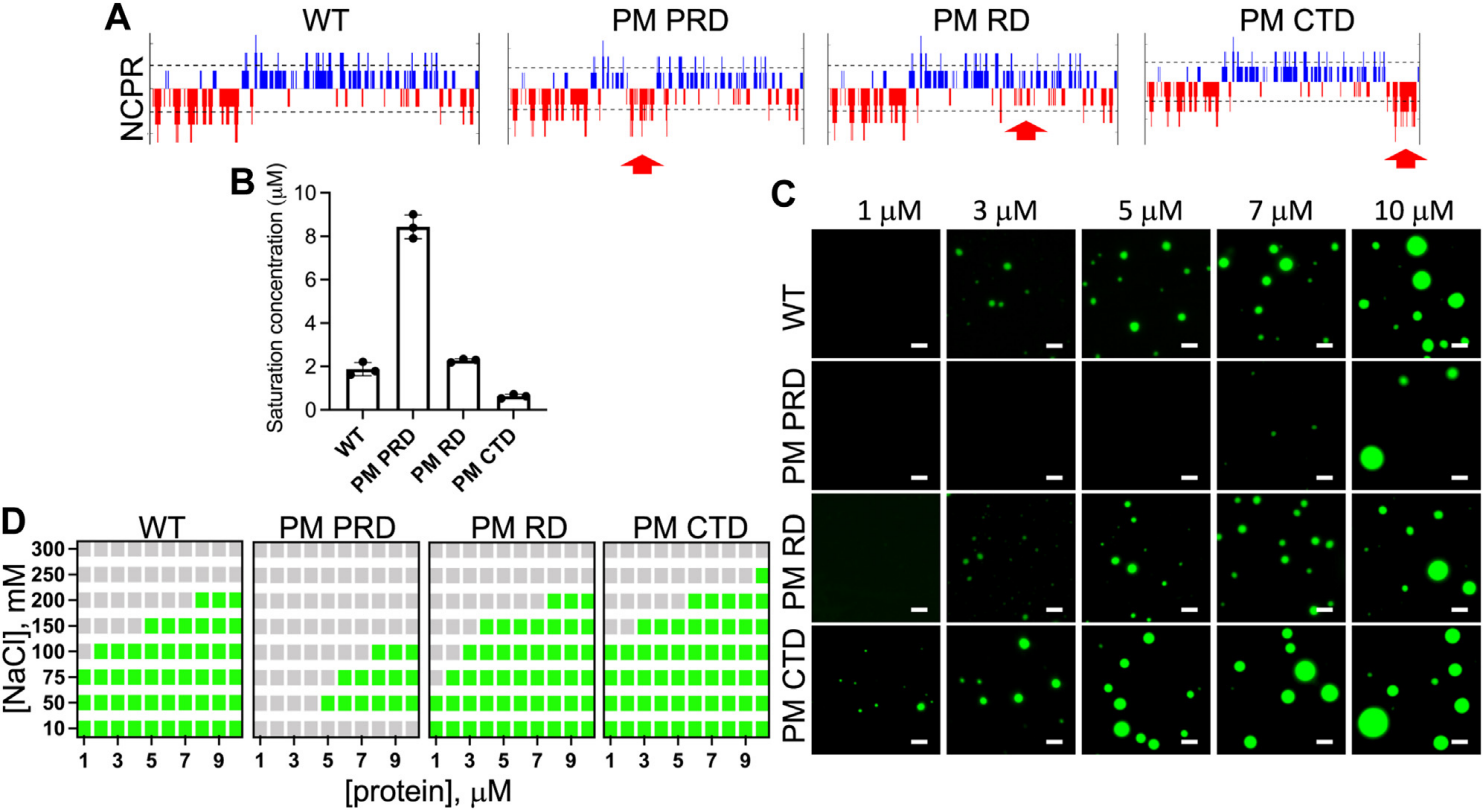
**LLPS propensity of the unmodified tau441 and the phosphomimetic tau441 variants.***A*, net charge per residue plots (NCPRs) of tau441 and its variants with the phosphomimetic substitutions in the proline-rich region (PM PRD), the repeat domain (PM RD), and the C-terminal domain (PM CTD). *Arrow* indicates the region where phosphomimetic substitutions were made. NCPR plots were generated using the algorithm available on the CIDER (49) webserver with a five-residue window. *B*, saturation concentrations for tau441 and the phosphomimetic variants. Error bars represent SD (n = 3). *C*, representative fluorescence microscopy images of tau441 and phosphomimetic variants at different protein concentrations (marked at the *top* of the panels). The images were obtained ∼10 min after sample preparation using 1:10 mixtures of Alexa Fluor 488–labeled and unlabeled proteins (scale bar represents 3 μm). *D*, protein concentration *versus* NaCl concentration phase diagrams. *Gray* and *green boxes* indicate the absence and presence of phase separation, respectively. Experiments were performed in 10 mM Hepes buffer (pH 7.4) containing 1 mM DTT, 2 mM EDTA, 10% PEG-10, and (unless indicated otherwise) 100 mM NaCl. Data were collected ∼10 min after sample preparation. LLPS, liquid-liquid phase separation.

Tau is characterized by highly polarized distribution of charges, where the N-terminal projection domain and CTD are negatively charged, and contrastingly, the PRD and RD are positively charged. This feature is especially important in the context of tau LLPS, as previous data suggest that condensation of tau is largely driven by intermolecular electrostatic interactions between these oppositely charged domains (20). We used this electrostatic model of tau LLPS as a starting point for designing phosphomimetic tau441 variants in which Ser/Thr residues found to be phosphorylated in AD (13, 14, 15) in different protein regions are substituted with Asp or Glu, respectively. Based on charge distribution plots (Fig. 1*A*), we hypothesized that phosphomimetic substitutions within the Pro-rich domain (PM PRD) (19 phosphorylation sites have been identified in this region in AD (13, 14, 15)) will have a deleterious effect on LLPS, as these substitutions reverse the overall charge in the middle region of tau from positive to negative, diminishing the potential for attractive intermolecular electrostatic interactions (Fig. 1*A* and Table 1). In contrast, phosphomimetic substitutions at 12 AD-related phosphorylation sites within the C-terminal 369-441 domain (PM CTD) (13, 14, 15) (which at neutral pH has net charge close to zero) are expected to increase the LLPS propensity for tau441, as these substitutions will create a negatively charged C-terminal “pole” that, akin to the N-terminal negatively charged region, could also engage in attractive intermolecular electrostatic interactions with the positively charged middle part of the protein (Fig. 1*A* and Table 1). Finally, phosphomimetic substitutions within the RD (PM RD) are anticipated to have a relatively modest impact on tau441 LLPS, since there are only four AD-relevant phosphorylation sites in this region (13, 14, 15) (Fig. 1*A* and Table 1).

**Table 1:** Details of phosphomimetic substitutions in different tau441 domains

Construct	Phosphomimetic substitutions	Overall charge	Charge in PRD	Charge in RD	Charge in CTD
WT		+ 2.9	+ 12.8	+ 10.2	- 0.9
PM PRD	T153E/T175E/T181E/S184D/S185D/S191D/S198D/S199D/S202D/T205E/S208D/S210D/T212E/S214D/T217E/T231E/S235D/S237D/S238D	- 16.1	- 6.2		
PM RD	S262D/S289D/S293D/S305D	- 1.1		+ 6.2	
PM CTD	S396D/S400D/T403E/S404D/S409D/S412D/S413D/S416D/S422D/T427E/S433E/S435E	- 9.1			- 12.9

### Probing the effect of phosphomimetic substitutions on tau441 LLPS *in vitro*

The capacity of different phosphomimetic tau441 variants to undergo LLPS was assessed by comparing their saturation concentrations (c_sat_) under the physiologically relevant buffer conditions in the presence of 10% PEG used to mimic macromolecular crowding in the cellular environment. Saturation concentration (*i.e.*, the concentration above which the system starts to phase separate) is a commonly used measure of protein LLPS propensity; it can be readily determined by turbidity measurements as a function of protein concentration (35). PM PRD of tau441 were found to increase the saturation concentration from ∼2 μM for the unmodified protein to ∼8 μM for the PM PRD variant (Figs. 1*B* and S1), indicating strongly reduced LLPS capacity of the latter protein. On the other hand, PM CTD led to a decrease in saturation concentration from ∼2 μM to ∼0.6 μM (Figs. 1*B* and S1), indicating an enhanced LLPS propensity of the PM CTD variant. Saturation concentration of tau441 with PM RD was not significantly different from that of unmodified protein. (Figs. 1*B* and S1). The presence of droplets for all tau variants studied at concentrations just above c_sat_ and their absence at concentrations just below c_sat_ has been further confirmed by fluorescence microscopy (Fig. 1*C*). It should be noted here that, in contrast to our finding, a previous study made a broad claim that phosphomimetic substitution of just three residues within the PRD (S199E/S202E/T205E) enhanced tau propensity for LLPS, even though this claim was in apparent conflict with authors’ own data showing that there was no statistically significant difference in c_sat_ (22).

The relative LLPS propensity of different phosphomimetic tau variants under a broader range of conditions was determined by constructing protein concentration *versus* NaCl concentration phase diagrams (Fig. 1*D*). These diagrams revealed that, compared to the unmodified tau441, the PM CTD variant was consistently more LLPS prone even at higher salt concentrations, whereas LLPS capacity of the PM PRD variant was consistently reduced under all conditions tested. As anticipated, phase diagram for the PM RD variant was very similar to that for the unmodified protein. Overall, these combined data support our hypothesis that phosphorylation patterns that enhance the polarity of charge distribution should promote tau441 LLPS, whereas those that diminish charge polarity should have an opposite effect.

Finally, to probe the involvement of interactions other than purely electrostatic in LLPS of phosphomimetic tau441 variants, we used 1,6-hexanediol, a compound known to inhibit LLPS of some proteins, presumably by disrupting hydrophobic and/or other nonionic interactions (36, 37). Similar to the unmodified protein (20), even at a concentration as high as 10%, 1,6-hexanediol was found to have very little, if any, effect on the capacity of all phosphomimetic variants tested to undergo LLPS (Fig. S2).

### Corecruitment of tau variants into droplets

**Figure 2:**
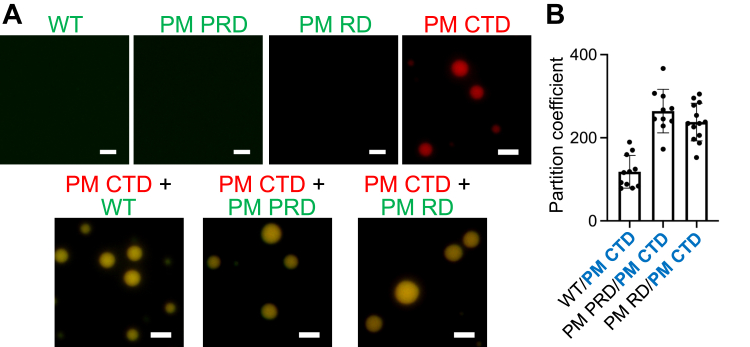
**Recruitment of tau variants with low LLPS propensity to droplets formed from PM CTD, the variant with high propensity for LLPS.***A*, (*top row*) representative fluorescence microscopy of the unmodified tau441 and the PM PRD and PM RD tau variants at 0.2 μM (*i.e.*, below saturation concentration of these proteins) and the PM CTD variant at 5 μM (*i.e.*, well above its saturation concentration); (*bottom row*) recruitment of the unmodified tau441 and the PM PRD and PM RD variants (0.2 μM in each case) to droplets formed by the PM CTD variant (5 μM). Unmodified tau441, the PM PRD, and PM RD variants were labeled with Alexa Fluor 488 (*green*), and the PM CTD variant was labeled with Alexa Fluor 594 (*red*). The ratio of labeled to unlabeled protein was 1:10 in each case. Scale bar represents 3 μm. *B*, partition coefficients for the recruitment of tau variants with low LLPS propensities (labeled in *black*) to the droplet phase of the PM CTD variant. These coefficients were calculated as the ratio of fluorescence intensity within the droplet and that in the dilute phase at protein concentrations as described for panel *A*. At least 10 droplets of each protein variant were analyzed. Error bars represent SD. Experiments were performed in 10 mM Hepes buffer (pH 7.4) containing 100 mM NaCl, 1 mM DTT, 2 mM EDTA, and 10% PEG-10. The microscopy images were obtained and fluorescence intensities within droplets measured ∼10 min after sample preparation. CTD, C-terminal domain; LLPS, liquid-liquid phase separation; PM PRD, phosphomimetic substitutions within the Pro-rich domain; PM CTD, phosphomimetic substitutions in the C-terminal domain; PM RD, phosphomimetic substitutions in the repeat domain; PRD, proline-rich domain; RD, pseudorepeat domain.

Next, we sought to determine whether tau variants with lower propensity for LLPS could be recruited to the droplets formed by the phosphomimetic variant with higher propensity for LLPS. To this end, we formed droplets of the PM CTD variant (which has a very low saturation concentration of 0.6 μM) and monitored the recruitment to these droplets of either unmodified tau441 or the PM PRD and PM RD variants, all at submicromolar concentrations (*i.e.*, well below their saturation concentrations) (Fig. 2*A*). Fluorescence microscopy images clearly demonstrated that all these tau variants with lower LLPS propensity were recruited into preformed PM CTD droplets. The efficiency of this recruitment was remarkably high, with the partition coefficient above 100 in each case (Fig. 2*B*). Furthermore, we found that the recruitment of lower LLPS propensity phosphomimetic tau variants to droplets formed by the PM CTD variant was not inhibited in the presence of 10% 1,6-hexanediol (Fig. S3), which suggests that this process is largely driven by electrostatic interactions. Importantly, no droplet formation was observed when the low and high LLPS propensity variants were mixed together at concentrations below their respective c_sat_ values, even when the cumulative concentration of both proteins exceeded c_sat_ for the high LLPS propensity variant (Fig. S4). Thus, the recruitment is not due to a synergistic action of both proteins in a dilute phase but, rather, occurs only when the higher LLPS propensity variant is already in the condensed phase.

**Figure 3:**
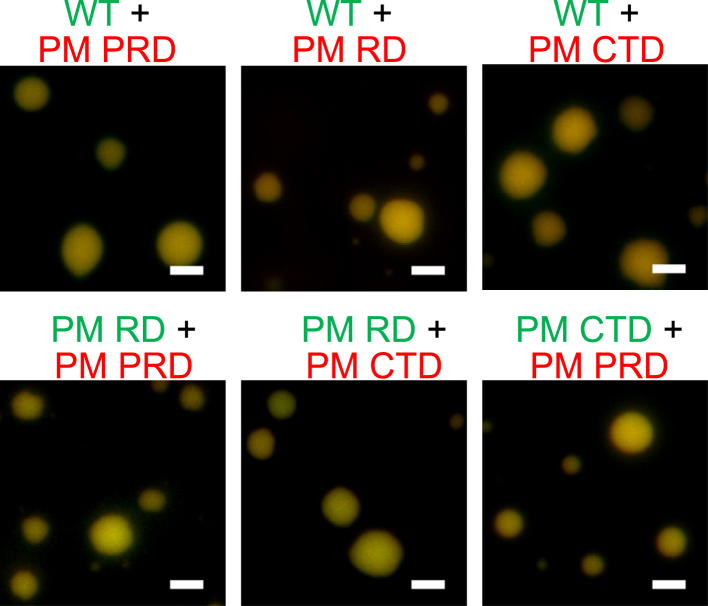
**Representative fluorescence microscopy images demonstrating colocalization and miscibility of the unmodified tau441 and the phosphomimetic variants within liquid droplets.** Proteins above their respective saturation concentrations (5 μM for unmodified tau441 and the PM RD and PM CTD variants; 10 μM for the PM PRD variant) were mixed in 10 mM Hepes buffer (pH 7.4) containing 100 mM NaCl, 1 mM DTT, 2 mM EDTA, and 10% PEG-10. Unmodified tau441 and the phosphomimetic variants were labeled with Alexa Fluor 594 (*red*) or Alexa Fluor 488 (*green*) as indicated at the *top* of individual panels. Colocalization within droplets resulted in *yellow* fluorescence. Images were obtained ∼10 min after sample preparation. Scale bars represent 3 μm. CTD, C-terminal domain; PM PRD, phosphomimetic substitutions within the Pro-rich domain; PM CTD, phosphomimetic substitutions in the C-terminal domain; PM RD, phosphomimetic substitutions in the repeat domain; PRD, proline-rich domain; RD, pseudorepeat domain.

While in some cases droplets formed by two different proteins are fully miscible (38, 39, 40), it is not uncommon for proteins to be immiscible within the condensed phase (*i.e.*, to form separate droplets that stay apart or to form droplets that colocalize inhomogenously) (41, 42, 43, 44). Therefore, we explored the miscibility properties of droplets formed by unmodified tau441 and its phosphomimetic variants. To this end, two-component mixtures of tau441 (labeled with green fluorophore) and any of its phosphomimetic variants (labeled with red fluorophore) as well as pairs of different phosphomimetic variants were prepared under the conditions where both proteins could independently undergo LLPS (*i.e.*, the concentration of each protein was above its saturation concentration). For each of these mixtures, freshly prepared individual droplets contained both proteins that appeared to be fully miscible in the condensed phase (Fig. 3). Remarkably, this miscibility was observed even after 18 h of incubation (Fig. S5).

### Probing the effect of phosphomimetic substitutions on the material properties of tau droplets

We next asked how phosphomimetic substitutions in different tau domains affect the material properties of the droplets. Fluorescence recovery after photobleaching (FRAP) experiments were employed to answer this question. In a previous report (29), we established that, in the absence of any polyanionic cofactors, droplets formed from tau441 or its pathogenic mutants such as P301L or ΔK280 tau do not significantly age with time, remaining highly dynamic for many hours after formation (29). However, significant aging of these droplets was observed in the presence of an anionic cofactor, heparin (29). This process was especially rapid for tau441 with a disease-related mutation such as P301L (29). Thus, the latter tau variant was chosen in the present study as a tractable experimental model for assessing the effect of phosphomimetic substitutions on time-dependent material properties of tau droplets.

**Figure 4:**
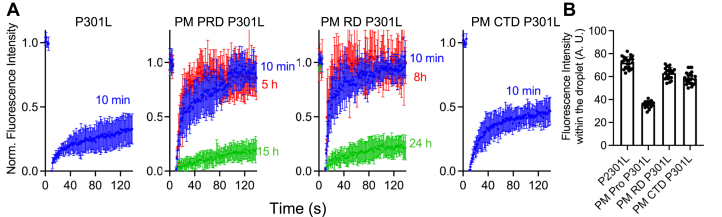
**The effect of phosphomimetic substitutions on time-dependent changes in material properties of droplets formed from P301L tau441 and its phosphomimetic variants.***A*, representative FRAP traces for droplets prepared from P301L tau441 and its phosphomimetic variants in the presence of heparin (protein to heparin molar ratio of 1:2). The concentration of proteins was 20 μM except the PM PRD variant, in which case it was 30 μM. The molar ratio of Alexa Fluor 488–labeled protein to unlabeled protein was 1:10 in each case. The FRAP traces were obtained at different time points after LLPS induction by the addition of 10% PEG-10. These time points are indicated by numbers next to the traces. Each trace represents the average of measurements for at least five droplets; error bars represent SD. *B,* relative fluorescence intensities within the droplets. Data were corrected for differences in the extent of labeling for different protein variants by normalization to the same value of absorbance at 488 nm. At least 17 droplets of each protein variant were analyzed.  Error bars represent SD. Experiments were performed in 10 mM Hepes buffer (pH 7.4) containing 100 mM NaCl, 1 mM DTT, 2 mM EDTA, and 10% PEG-10. FRAP, fluorescence recovery after photobleaching; LLPS, liquid-liquid phase separation; PRD, proline-rich domain; PM PRD, phosphomimetic substitutions within the Pro-rich domain.

Consistent with the previous report (29), unmodified P301L tau441 droplets incubated for 10 min after preparation exhibited reduced dynamicity, showing only ∼30% recovery of their initial fluorescence signal after photobleaching (Fig. 4*A*). In sharp contrast, droplets formed from the phosphomimetic PM PRD and PM RD variants of P301L tau441 exhibited rapid fluorescence recovery (90–100% within 120 s) after 10 min and even several hours of incubation, indicating highly dynamic nature of these proteins within the condensed phase (Fig. 4*A*). These droplets still aged upon longer incubation times, however, with PM PRD droplets showing little fluorescence recovery after 15 h of incubation. This apparent transition from a liquid-like to a rigid-like state was even slower for droplets formed from the PM RD variant, in which case residual fluorescence recovery was observed even after 24 h incubation (Fig. 4*A*). PM CTD of P301L tau441 were found to have a much smaller effect on droplet material properties, even though a measurable decrease in droplet aging rate compared to that of the unmodified protein (∼45% *versus* ∼30% fluorescence recovery for droplets incubated for 10 min) was also observed in this case. (Fig. 4*A*). Taken together, these data indicate that phosphomimetic substitutions studied slow down aging of tau condensates and their transition to a rigid state, with the degree of this effect strongly depending on the location of these substitutions.

One potential factor that could contribute to difference in time-dependent material properties of droplets formed by different phosphomimetic tau variants is the concentration of these proteins in the condensed phase. This can be readily assessed by measuring the fluorescence intensity within each droplet type and normalizing these data to the same molar ratio of the fluorescent dye to total protein. Using this approach, we found that the concentration of the PM PRD tau variant within droplets is significantly lower than other tau variants studied, while the differences between other variants (including the unmodified tau) are very modest (Fig. 4*B*).

### Probing the effect of phosphomimetic substitutions on tau fibrillation under the condition of LLPS

Next, we sought to determine how phosphomimetic substitutions affect the ability of P301L tau441 to aggregate into amyloid fibrils under LLPS conditions. To this end, we monitored fibril formation of P301L tau441 and its phosphomimetic variants (5 μM each) in the presence of 10 μM heparin and 10% PEG-10 using a thioflavin T (ThT) fluorescence assay. Under these conditions, P301L tau441 and its phosphomimetic PM RD and PM CTD variants undergo robust LLPS, with c_sat_ in the submicromolar range (Fig. S6). In the absence of LLPS, fibrillation of these tau variants at concentrations slightly below c_sat_ was very slow, as indicated by the lack of significant ThT fluorescence increase up to at least 46 h (Fig. S7). Thus, when experiments were performed under LLPS conditions (*i.e.*, above c_sat_), we could selectively monitor protein fibrillation within the droplets (29). No such selective monitoring could be done, however, for the PM PRD variant, in which case the saturation concentration is much higher and protein aggregation both inside and outside the droplets could potentially contribute to the ThT fluorescence signal.

**Figure 5:**
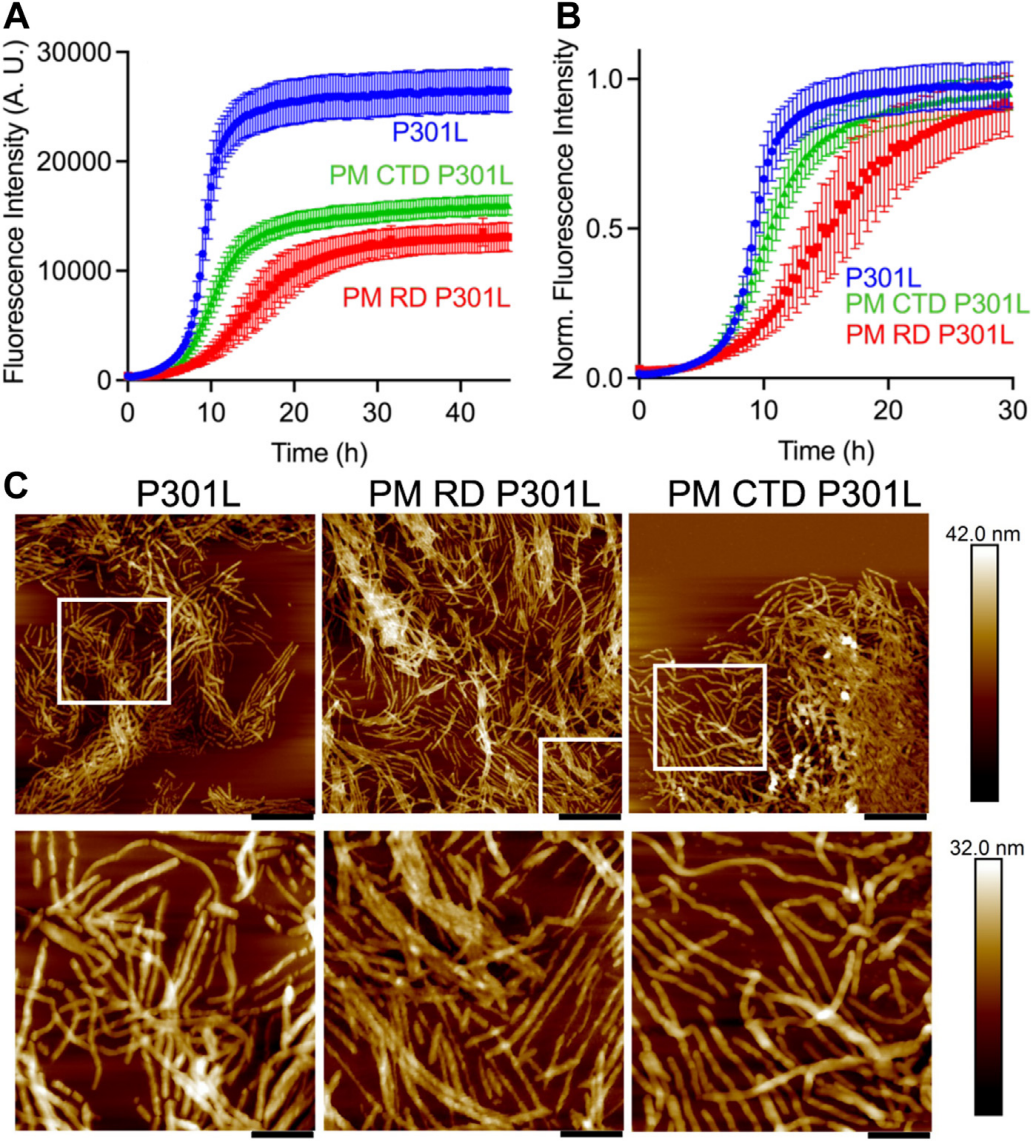
**The effect of phosphomimetic substitutions on fibrillation kinetics of P301L tau441 under the condition of LLPS.***A*, ThT fluorescence traces for P301L tau441 and its phosphomimetic variants (5 μM protein, 10 μM heparin in each case) under the condition of LLPS. Error bars represent SD (n = 6). *B*, the same traces as in panel *A* after normalization for ThT fluorescence differences at the end of the growth phase. *C*, representative atomic force microscopy images for P301L tau441 and its phosphomimetic variants obtained 30 h after start of the fibrillation reaction. *Top row*: scale bars represent 660 nm; *bottom row*: higher magnification of selected areas in images shown in the *top row*, scale bars represent 220 nm. Fibrillation reactions were carried out in 10 mM Hepes buffer (pH 7.4) containing 100 mM NaCl, 1 mM TCEP, 2 mM EDTA, and 10% PEG-10. LLPS, liquid-liquid phase separation; ThT, thioflavin T.

The ThT fluorescence curves for fibrillation of P301L tau441 and its highly LLPS-prone PM RD and PM CTD variants are shown in Figure 5*A* (raw data) and Figure 5*B* (same data after normalization for differences in the final intensities of ThT fluorescence). These data reveal that PM RD significantly decrease the rate of P301L tau fibrillation within the droplets compared to that of unmodified P301L tau (reaction half-times of 15 and 9 h, respectively), whereas phosphomimetic substitutions in the C-terminal region have very little effect on the fibrillation rate (reaction half-time of 11 h). It should also be noted that the intensity of ThT fluorescence at the end of the growth phase is substantially higher for the unmodified protein than that for the phosphomimetic variants (Fig. 5*A*). This likely reflects lower binding affinity of the negatively charged ThT to fibrils formed from the phosphomimetic variants, even though potential structural differences between the fibrils could also contribute to fluorescence intensity differences.

The morphology of aggregates formed under the conditions of LLPS was examined by atomic force microscopy (AFM). The images obtained at the end of the growth phase after 30 h of incubation are shown in Figure 5*C*. For both the P301L tau441 and the phosphomimetic variants, regions of densely packed fibrils surrounded by regions lacking fibrillar structures were observed, further indicating that fibrils were formed within the droplets.

## Discussion

The role of phosphorylation in tau LLPS is somewhat controversial. An early study using protein expressed in insect cells asserted that phosphorylation is a prerequisite for tau LLPS (18). This, however, has not been confirmed in subsequent reports from several laboratories, as these studies consistently demonstrated high capacity of nonphosphorylated protein to condense into liquid droplets (19, 20, 22). Another controversy exists regarding the mechanism of tau LLPS and the role of different interaction types in this reaction. While some studies suggested an important role of hydrophobic interactions (45), most other studies point to a largely, if not purely, electrostatic mechanism of tau LLPS (20, 22). The latter mechanism is strongly supported by studies with a series of deletion tau variants (20, 22) and variants with pathogenic mutations (29), which led us to the hypothesis that LLPS of full-length tau under physiologically relevant buffer conditions is largely driven by attractive intermolecular interactions between the negatively charged N-terminal and positively charged PRD and RD (20).

In an attempt to resolve these controversies and gain insight into the mechanism by which phosphorylation could modulate tau LLPS, here we performed systematic studies by introducing phosphomimetic substitutions of Ser/Thr residues with negatively charged Asp/Glu residues in different regions of the protein. Phosphomimetic substitutions are not a perfect model for phosphorylation, as phosphate groups are bulkier and can carry larger charge at physiological pH. However, the advantage of this commonly used approach is that it allows preparation of a homogeneous population of protein selectively modified at desired sites. The finding that substitutions that increase the overall polarization of charges on tau molecules promote LLPS, and those that decrease charge polarization have an opposite effect, provides a strong support for the electrostatic model of tau LLPS and the notion that this reaction is driven by attractive electrostatic intermolecular interactions between the oppositely charged protein regions as described in the preceding paragraph.

Our simple phosphomimetic system cannot recapitulate the complexity of tau phosphorylation in a cellular environment, where many subpopulations of tau molecules with different phosphorylation patterns are present (see the Introduction). Nevertheless, our finding that phosphorylation patterns that increase the overall charge polarization in tau should increase the propensity for LLPS has potentially important implications, as the proportion of tau molecules with such LLPS-promoting phosphorylation patterns is likely to be quite substantial. In this context, we attach particular significance to the present observation that once these highly LLPS-prone tau molecules form liquid droplets, these condensates are able to efficiently recruit tau molecules with phosphorylation patterns that impart lower intrinsic propensity for LLPS. The apparent lack of sensitivity to hexanediol suggests that, akin to *de novo* droplet formation, this recruitment is also largely driven by electrostatic interactions. The identities of the specific charged residues and/or protein segments involved in these electrostatic interactions are at present unknown. Nevertheless, it is tempting to speculate that the recruitment process may be mediated by conformational transition(s) of high LLPS propensity tau variants already within the droplets that result in the exposure of specific clusters of charged residues, thereby facilitating attractive electrostatic interactions between these variants and the low LLPS propensity variants to be recruited to the droplets. Even though further studies are needed to probe this issue, the above hypothesis is generally consistent with the observation that tau in the condensed phase adopts a more extended conformation than it does in the dilute phase (31). Questions regarding the mechanism of the recruitment process notwithstanding the present observations suggest that phosphorylation could potentially provide a powerful regulatory mechanism of tau condensation in a cellular environment.

The present data also reveal that, apart from modulating the propensity of tau for LLPS, phosphomimetic substitutions (and likely also authentic phosphorylation) influence the material properties of tau within the condensed phase, generally slowing down the process of droplet aging and their transition to a more rigid state. This is of potential pathophysiological consequence, as transient crosslinks and/or oligomers formed during droplet aging may be direct precursors of fibrillar aggregates. Our previous study with tau variants carrying pathogenic mutations within the repeat domain suggested that the same types of interactions are likely involved in droplet aging and fibril formation (29). However, the present data indicate that, at least for the phosphomimetic tau variants, the relationship between droplet aging and protein fibrillation within the droplets may be more complex. For example, while droplets formed from unmodified P301L tau and the PM CTD variant show substantial loss of dynamicity already within 10 min of incubation (*i.e.*, at very early stages of the lag phase of the fibrillation reaction), those formed from the PM RD variant remain highly dynamic even at the end of the lag phase. This suggests that different mechanisms may be involved in aging of droplets formed from different phosphomimetic tau variants. It should be noted that experiments on droplet aging and fibrillations were performed in the presence of an anionic cofactor heparin. Even though heparin is not present in neurons, it is widely used as a readily available cofactor that mimics the effects of heparan sulfate, a polyanion identified as a potentially key player in tau aggregation in brain (46).

Finally, it should be noted that while most studies emphasize the crucial role of weak side chain–side chain interactions in LLPS of proteins, a recent study postulates that this process is largely driven by the backbone interactions similar to those responsible for β-sheet formation in protein fibrillation reaction (47). The present data argue against the latter scenario as a generally valid model, strongly suggesting that condensation of tau into highly dynamic liquid droplets and subsequent fibrillation of this protein are driven by different types of intermolecular interactions.

## Experimental procedures

### Expression, purification, and labeling of tau variants

Genes encoding phosphomimetic tau variants were synthesized by Genscript and subcloned into the pET-15b vector using the CloneEZ technique. PM PRD and PM CTD genes contained N-terminal His-tag and HRV 3C cleavage site sequences. Unmodified tau441 was purified as described previously (20), and a similar protocol was used for purification of the PM RD variant, except that Mes buffer pH 5.5 was used instead of Mes buffer pH 6.8. PM PRD and PM CTD were purified on an immobilized nickel-affinity column (Ni-NTA Fast Flow, Qiagen) and dialyzed against 10 mM Hepes, 20 mM NaCl, 2 mM DTT, 0.1 mM PMSF, pH 7.4. This was followed by further purification on an anion-exchange column (Mono-Q, GE HealthCare). Proteins were eluted using a linear gradient of NaCl, and fractions containing tau were pooled. Proteins were dialyzed against buffer (10 mM Hepes, 100 mM NaCl, 2 mM DTT, pH 7.4). The His tag was cleaved by incubating overnight at room temperature with HRV 3C protease (Pierce; 2 units/mg protein). HRV 3C protease was then captured using glutathione agarose beads, and free His tag was removed by size-exclusion chromatography as described previously (20). The cleaved proteins contained three additional N-terminal residues (GPH). Protein concentration was measured by a reducing agent–compatible bicinchoninic acid protein assay (Thermo Fisher Scientific). Proteins were fluorescently labeled with Alexa fluor 488 or 594 Succinimidyl Ester (Invitrogen) as described previously (20).

### Turbidity measurements

Sample turbidity (absorbance at 400 nm) was measured at 37 °C using the Tecan Spark multimode microplate reader with Te-CoolTM active temperature regulation. This was done in 10 mM Hepes buffer (pH 7.4) containing 100 mM NaCl, 1 mM DTT, 2 mM EDTA, and freshly prepared PEG 10,000 (PEG-10) (Sigma-Aldrich).

### Fluorescence microscopy imaging

Droplets of tau441 and its phosphomimetic variants (formed in the same buffer as used for turbidity measurements) were visualized by fluorescence microscopy using Alexa Fluor 488– or Alexa Fluor 594–labeled tau variants as described previously (29). The ratio of labeled to unlabeled protein was 1:10.

### FRAP experiments

Droplets were prepared as described above for fluorescence microscopy imaging experiments. FRAP measurements were performed on a Leica HyVolution SP8 confocal microscope as described previously (29). Individual fluorescence traces were normalized to maximal prebleach and minimal postbleach intensities.

### ThT fluorescence assay

The fibrillation reactions were carried out at 37 °C under quiescent conditions and monitored by the ThT assay as described previously (29). This was done in 10 mM Hepes buffer (pH 7.4) containing 1 mM Tris(2-carboxyethyl)phosphine, 2 mM EDTA, 100 mM NaCl, 0.02% sodium azide, 20 μM ThT, 10% PEG-10, and low molecular weight heparin (4.48 kDa; European Pharmacopoeia Reference Standard; protein to heparin molar ratio of 1:2). Half-times of the reactions were determined from the experimental data fitted to the sigmoidal function.

### AFM imaging

AFM imaging was performed as described previously (29, 48).

## Data availability

All data are contained within this article and the supporting information.

## Supporting information

This article contains supporting information.

## Conflict of interest

The authors declare that they have no known competing financial interests or personal relationships that could have appeared to influence the work reported in this paper.
