# Supplementary material for: Domain-specific modulatory effects of phosphomimetic substitutions on liquid-liquid phase separation of tau protein
Source: J Biol Chem. 2023 Apr 17;299(6):104722. doi: 10.1016/j.jbc.2023.104722 (PMC10199205; doi:10.1016/j.jbc.2023.104722)
Supplement: Supporting information [file mmc1.pdf]

Supporting information for

Domain-specific modulatory effects of phosphomimetic substitutions on liquid-liquid phase separation of tau protein

Solomiia Boyko and Witold K. Surewicz*

Department of Physiology and Biophysics, Case Western Reserve University, Cleveland, Ohio 44106

*Corresponding author. Email: wks3@case.edu

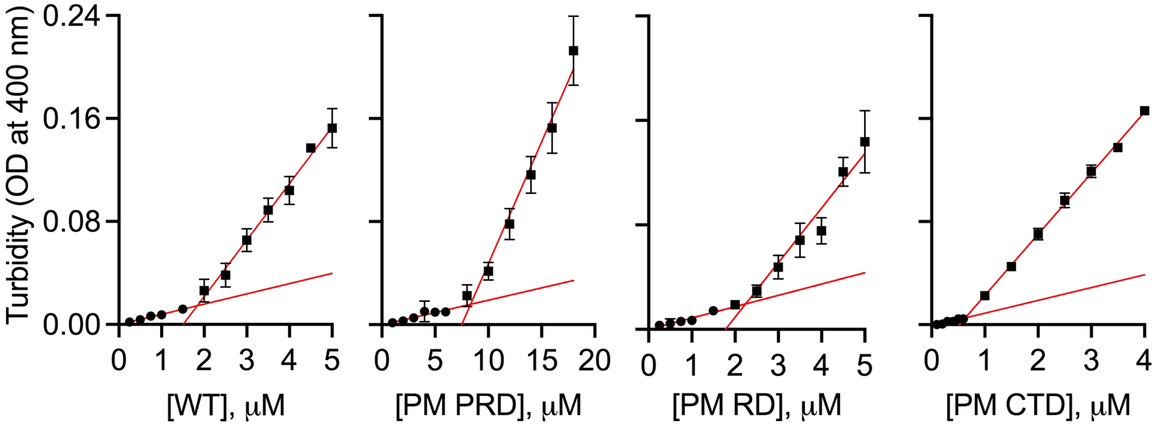

Figure S1. Determination of saturation concentrations (c_{sat}) for tau441 and its phosphomimetic variants. Turbidity (OD at 400 nm) for each variant was measured as a function of protein concentration. The concentrations at which the two straight lines drawn through experimental data points intersect correspond to c_{sat}. Error bars represent SD (n=3). Experiments were performed in 10 mM HEPES buffer (pH 7.4) containing 100 mM NaCl, 1 mM DTT, and 2 mM EDTA and 10% PEG-10. Data were collected ~10 min after sample preparation.

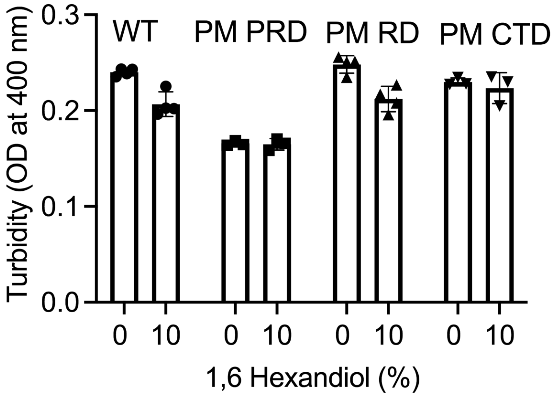

Figure S2. The effect of 1,6-hexanediol on LLPS of tau441 and its phosphomimetic variants as assessed by turbidimetric measurements. Experiments were performed in 10 mM HEPES buffer (pH 7.4) containing 100 mM NaCl, 1 mM DTT, 2 mM EDTA and 14% PEG-10. Error bars represent SD (n >3). Data were collected ~10 min after sample preparation.

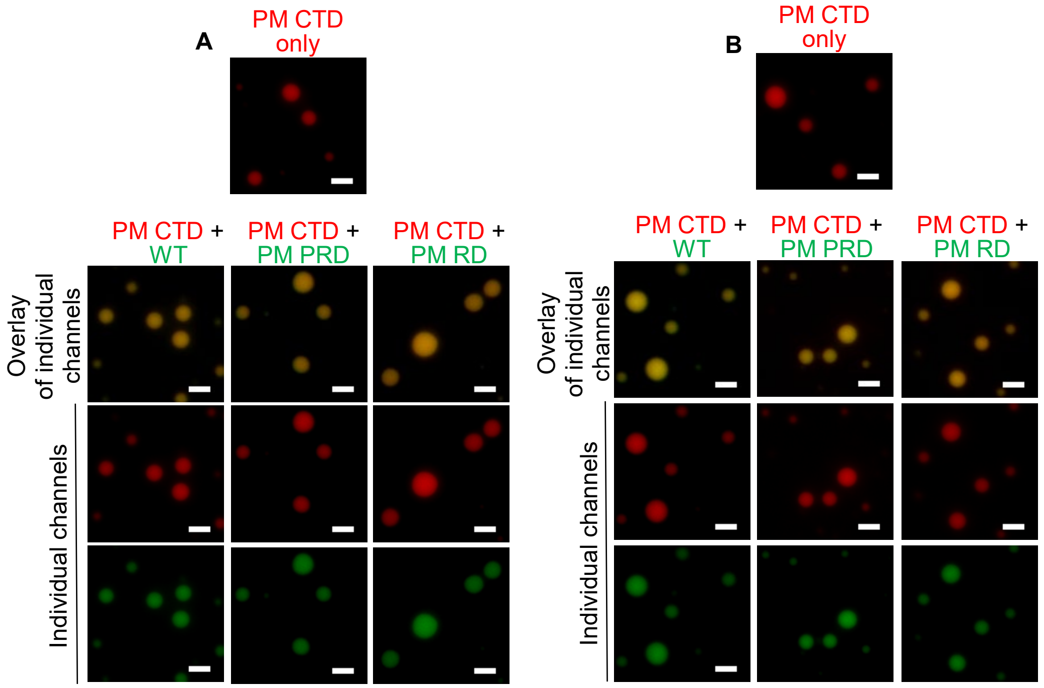

Figure S3. Recruitment of tau variants with lower LLPS propensity (unmodified tau441, PM PRD, and PM RD variants) to droplets formed from PM CTD, the variant with high LLPS propensity in the absence (*A*) or presence of 1,6-hexanediol (*B*). The top row in each panel represents the PM CTD variant only. The concentration of the unmodified tau441 and the PM PRD and PM RD variants was 0.2 μM and that of the PM CTD variant was 5 μM). Unmodified tau441, PM PRD, and PM RD variants were labeled with Alexa Fluor 488 (green), and the PM CTD variant was labeled with Alexa Fluor 594 (red). The ratio of labeled to unlabeled protein was 1:10 in each case. Scale bar, 3 μm. Experiments were performed in 10 mM HEPES buffer (pH 7.4) containing 100 mM NaCl, 1 mM DTT, 2 mM EDTA and 10% PEG-10. Images were obtained ~10 min after sample preparation. The control PM CTD only image in panel *A* is same as the PM CTD image in Figure 2A. Furthermore, overlay images in panel *A* are same as those in the bottom row of Figure 2.

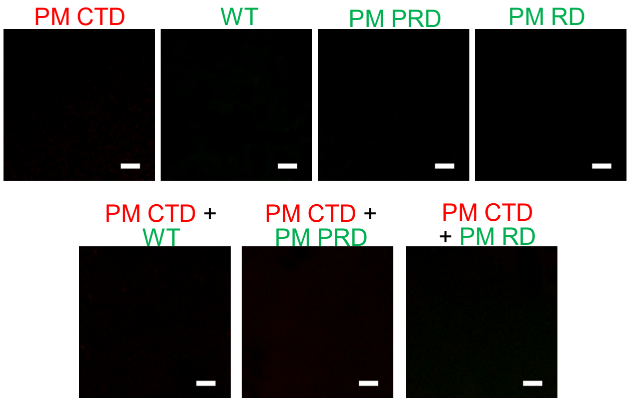

Figure S4. Fluorescence microscopy images demonstrating that that the mixtures of PM CTD tau, a variant with high LLPS propensity, and other tau variants studied do not undergo LLPS at concentrations below their respective c_{sat} values, even when the combined concentration of both proteins is above c_{sat} of the PM CTD variant (0.6 μM). *Top row*, individual proteins at a concentration of 1 μM (unmodified tau441 and the PM PRD and PM RD variants) and 0.2 μM (the PM CTD variant), i.e., below saturation concentrations of these proteins. *Bottom row*, mixtures of the PM CTD tau variant (0.2 μM) with unmodified tau441 and the PM PRD and PM RD variants (1 μM in each case). Unmodified tau441, the PM PRD and PM RD variants were labeled with Alexa Fluor 488 (green), and the PM CTD variant was labeled with Alexa Fluor 594 (red). The ratio of labeled to unlabeled protein was 1:10 in each case. Scale bar, 3 μm. Experiments were performed in 10 mM HEPES buffer (pH 7.4) containing 100 mM NaCl, 1 mM DTT, 2 mM EDTA and 10% PEG-10. Images were obtained ~10 min after sample preparation. These fluorescence images were acquired using identical microscope settings as those in experiments where LLPS was observed.

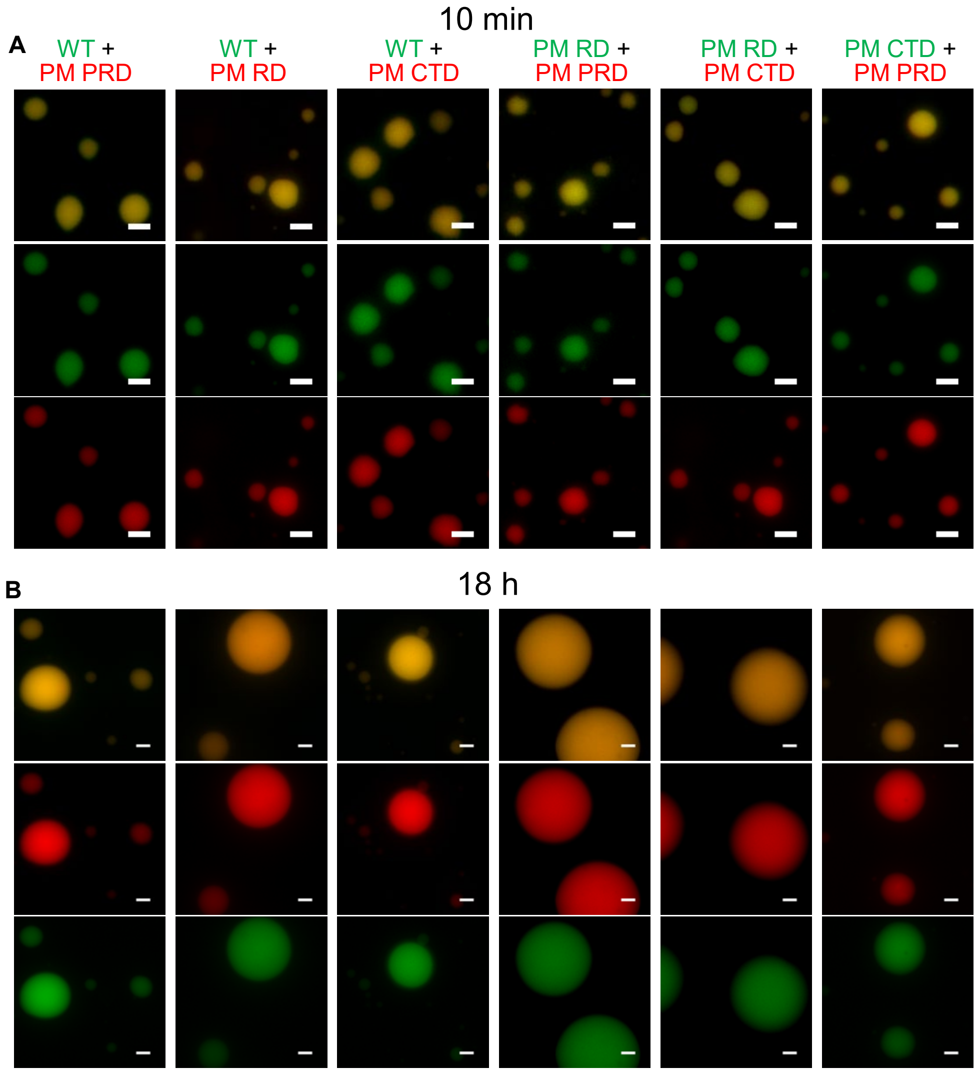

Figure S5. Representative fluorescence microscopy images demonstrating colocalization and miscibility of the unmodified tau441 and the phosphomimetic variants within liquid droplets 10 minutes (*A*) and 18 h (*B*) after droplet formation. Proteins above their respective saturation concentrations (5 µM for the unmodified tau441 and the PM RD and PM CTD variants; 10 µM for the PM PRD variant) were mixed in 10 mM HEPES buffer (pH 7.4) containing 100 mM NaCl, 1 mM DTT, 2 mM EDTA and 10% PEG-10. Unmodified tau441 and the phosphomimetic variants were labeled with Alexa Fluor 594 (red) or Alexa Fluor 488 (green) as indicated at the top of individual panels. Colocalization within droplets resulted in yellow fluorescence. Scale bars, 3 µm (panel *A*); 10 µm (panel *B*). Overlay images in the top row of panel *A* are same as those shown in Figure 3.

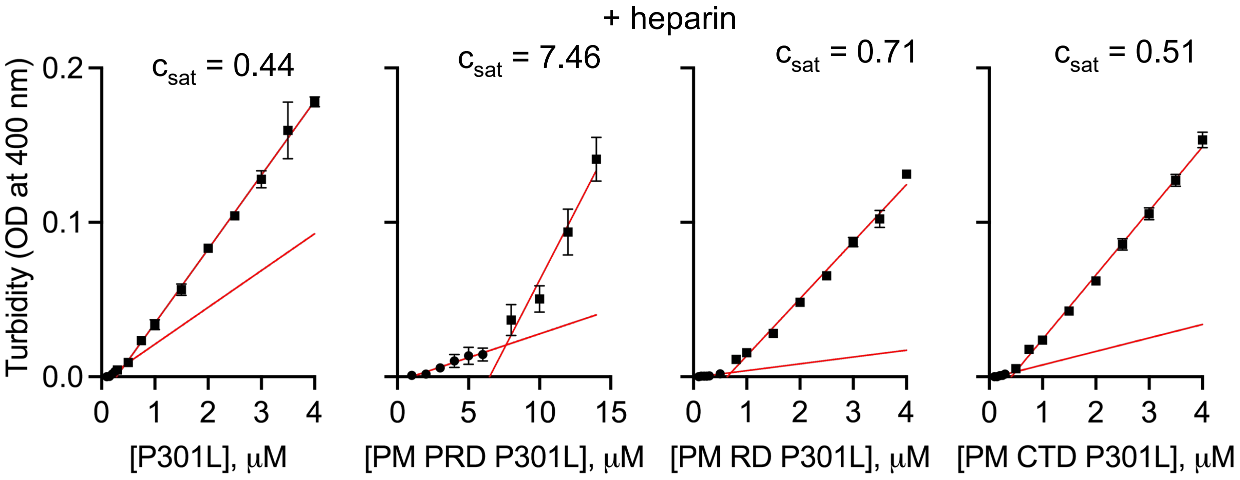

Figure S6. Determination of saturation concentrations (c_{sat}) for tau441 and its phosphomimetic variants in the presence of heparin. *A,* Turbidity (OD at 400 nm) for each variant in the presence of heparin (tau to heparin molar ratio of 1:2) was monitored as a function of protein concentration. The concentrations at which the two straight lines drawn through experimental data points intersect correspond to c_{sat}. Experiments were performed in 10 mM HEPES buffer (pH 7.4) containing 100 mM NaCl, 1 mM DTT, 2 mM EDTA and 10% PEG-10. Error bars represent SD (n=3). Data were collected ~10 min after sample preparation.

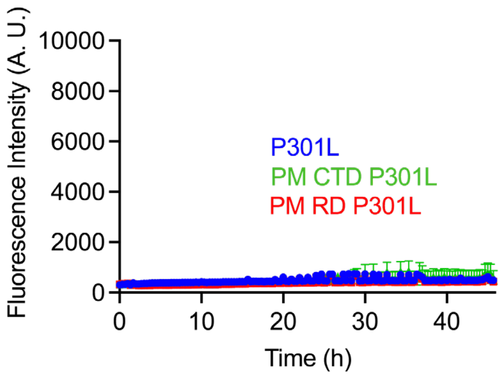

Figure S7. ThT fluorescence traces for the unmodified P301L tau441 (0.3 μM), PM RD P301L tau441 (0.5 μM), and PM CTD P301L tau441 (0.3 μM). At these concentrations all proteins are below their saturation concentrations. Experiments were performed in 10 mM HEPES buffer (pH 7.4) containing 100 mM NaCl, 1 mM TCEP, 2 mM EDTA and 10% PEG-10 and heparin (protein to heparin molar ratio of 1:2 in each case). Error bars represent SD (n = 6).
